# Automatic detection of procedural knowledge in robotic-assisted surgical texts

**DOI:** 10.1007/s11548-021-02370-9

**Published:** 2021-04-22

**Authors:** Marco Bombieri, Marco Rospocher, Diego Dall’Alba, Paolo Fiorini

**Affiliations:** University of Verona, Verona, Italy

**Keywords:** Embeddings, Text classification, Deep learning, Transformers, Procedural knowledge, Surgical data science

## Abstract

**Purpose:**

The automatic extraction of knowledge about intervention execution from surgical manuals would be of the utmost importance to develop expert surgical systems and assistants. In this work we assess the feasibility of automatically identifying the sentences of a surgical intervention text containing procedural information, a subtask of the broader goal of extracting intervention workflows from surgical manuals.

**Methods:**

We frame the problem as a binary classification task. We first introduce a new public dataset of 1958 sentences from robotic surgery texts, manually annotated as procedural or non-procedural. We then apply different classification methods, from classical machine learning algorithms, to more recent neural-network approaches and classification methods exploiting transformers (e.g., BERT, ClinicalBERT). We also analyze the benefits of applying balancing techniques to the dataset.

**Results:**

The architectures based on neural-networks fed with FastText’s embeddings and the one based on ClinicalBERT outperform all the tested methods, empirically confirming the feasibility of the task. Adopting balancing techniques does not lead to substantial improvements in classification.

**Conclusion:**

This is the first work experimenting with machine / deep learning algorithms for automatically identifying procedural sentences in surgical texts. It also introduces the first public dataset that can be used for benchmarking different classification methods for the task.

## Introduction

Thousands of different types of surgical procedures are performed daily in hospitals around the world. These procedures are typically described in detail in written resources (e.g., books, manuals, academic papers, online resources), abundantly available nowadays, that are used by medical students to acquire or refine their knowledge. The description of a procedure conveys the so-called *procedural knowledge* i.e. the knowledge possessed by an intelligent agent (in surgery, a surgeon or a surgical robot) able to perform a task (a surgical intervention). Typically, the description of a procedure details how to perform the surgical intervention, which anatomical parts to operate, and which tools to use. The standard workflow is generally divided into phases and steps organized into several branches. Each branch describes a particular condition that might occur, such as unexpected patient vital parameters or particular anatomical configurations, and the action flow to follow to handle that specific situation.

Extracting structured workflows from surgical textual resources would be beneficial both for the development of expert surgical systems and for the assistance to the doctor during surgery. However, the manual extraction of these workflows requires substantial human effort and expertise, hindering its application at scale. In our work we are interested in tackling the extraction of structured workflows from texts with automatic methods. In particular, the overall research objective can be split into two main steps. First, *procedural sentences* i.e., sentences containing procedural knowledge, are recognized in a text; in fact, real-world documents describing surgical processes usually include also descriptive information, which is not useful to extract the workflow. Then, the recognized sentences are used to extract knowledge and model the procedure workflow.

This paper presents our novel contribution to address the first of the above steps. To the best of our knowledge, no previous works investigated the *automatic* identification of procedural sentences in surgical texts. We tackle this problem by applying different machine learning (ML) and deep learning (DL) algorithms. In addition to consolidated ML approaches available in literature and tested in other domains, we experiment with the FastText Classifier, since it allows to achieve state-of-the-art performance in numerous text classification tasks. Moreover, we investigate the use of the subword-enriched word embeddings returned by FastText as features for a one-Dimensional Convolutional Neural-Network (1D-CNN) and a Bidirectional Long Short-Term Memory (Bi-LSTM) Neural-Network (NN). Finally, we test Transformers-based classification methods, fine-tuning some pre-trained language models for the considered task.

To train and benchmark all these approaches, we introduce a novel surgical textual dataset, consisting of sentences from surgical texts that we manually annotate as procedural or non procedural.

In summary, the contribution of the paper is threefold:

- the proposal to address the detection of procedural knowledge in surgical texts as a sentence classification task;
- a novel, publicly-available, manually-annotated surgical text dataset for benchmarking classification methods;
- a preliminary assessment on this dataset of various state-of-the-art classification methods.

The paper is organized as follows: in Sect. 2, we briefly describe relevant methods available in literature for procedural knowledge detection in other domains and for supervised text classification; in Sect. 3, we describe the characteristics of the contributed dataset, the preprocessing techniques applied, and methods used for the detection of procedural sentences. In Sect. 4, we present and discuss the results, while in Sect. 5, we summarize the work done and present future works.

## State of the art

To the best of our knowledge, no works have tackled so far the problem of detecting procedural sentences in surgical documents. However, approaches for detecting procedural sentences have been proposed in other domains and applied to typologies of textual content substantially different than the description of a surgical procedure, such as repair instructions [17, 27, 30], technical support documentation [2, 8, 17], instructions for nanomaterials’ synthesis [28], cooking recipes [17, 30] and medical abstracts [24].

In [2], the authors tackle the problem of procedural knowledge detection in technical documentation as a classification task. They use a linear Support Vector Machine (SVM) exploiting both linguistic (usage of imperative, declarative, conditional or passive form) and structural (e.g., section/subsection organization, bulleted-list usage) features, showing that both of them contribute to improve performances.

The authors of [17] address the problem of identifying sentences mentioning actions in cooking recipes and maintenance manuals, exploiting a CNN fed with word embeddings. Classification (“relevant”, “irrelevant”) of recipe (for nanomaterials’ synthesis) sentences is investigated also in [28], where the authors use a Naïve Bayes classifier fed with features such as word counts, TF-IDF (Term Frequency-nverse Document Frequency) and N-grams.

In [27], the authors pursue the detection of repair instructions in user-generated text from automotive web communities. Various features (bag-of-words, bag-of-bigrams, post length, readability index), including structural ones (repair instructions are often provided as bulleted or numbered lists) are fed to several ML methods, from classical ones (e.g., Random Forest) to Neural-Networks (single and multilayer perceptrons).

In [8], an SVM is applied for detecting procedural sentences in technical support documentation, where procedures are typically described using lists. Besides traditional features, such as TF-IDF, the authors show the effectiveness of exploiting also information on the list type, contextual features (e.g., sentences introducing a list), and the usage of imperatives.

The authors of [24] address the detection of procedural knowledge in MEDLINE abstracts. In their work, procedural knowledge is defined as a set of *unit procedures* (each consisting of a *Target*, *Action* and *Method*) organized for solving a specific purpose. The proposed solution works in two steps. First, SVMs and Conditional Random Fields (CRFs) are combined for detecting sentences (purpose/solution) that may contain unit procedures, feeding them with content (unigrams and bigrams), position (sentence number in the abstract), neighbor (content features of nearby sentences) and ontological features (usage of terms from reference vocabularies). Then, sequence labeling with CRFs is performed to identify the components of unit procedures.

Finally, the authors of [30] address the extraction of procedural knowledge from structured instructional texts. First, they partition sentences into related segments, from which finite-state grammars are applied to extract procedural elements. Next, basic rule-based reasoning is applied to resolve certain types of omissions and ambiguities in instructions.

While all these works address the detection of procedural knowledge from written text, we remark that the proposed methods have been applied on typologies of textual content substantially different from the description of a surgical procedure. Troubleshooting and product documentation, cocking recipes, maintenance manuals, and repair instructions differ from descriptions of surgical procedures both on the terminological/language level and the structural one: typically these kinds of texts are structurally organized, frequently using numbered/bulleted lists—a characteristic effectively exploited as feature in many of the discussed approaches—while no established standard way to describe a surgical procedure exists. In addition, surgical interventions are mainly presented in a prose-like style. Indeed, the scenario where structural features cannot be exploited is considered more challenging to tackle (c.f. [8]).

Furthermore, all the aforementioned approaches have been applied on domains that are substantially different from the surgical one. In this regards, the closest work is [24]: however, MEDLINE abstracts are substantially different from intervention descriptions (e.g., MEDLINE abstracts are typically semantically divided into blocks such as Objective, Background, Methods, etc.), and the goal of the authors is to identify (a few) methodological sentences among an abstract text, while our goal is identify all the sentences in a intervention procedure description that detail some surgical action performed.

## Proposed method

In this section, we first describe the collected dataset and then we detail our processing approach. We remark that our goal is not to propose a new state-of-the-art method for text classification but to assess whether the automatic classification of procedural knowledge in surgical written texts (never studied in literature before) can be effectively solved with ML or DL techniques for text classification.

### Dataset

In order to train and test a supervised classification approach to automatically identify procedural sentences, a dataset of sentences labeled as procedural/non-procedural is needed. Given the lack of such a dataset in the literature, we manually constructed and annotated a new dataset, called SPKS (Surgical Procedural Knowledge Sentences)^1^ composed by 1958 sentences (37022 words—3999 unique words) from a recent surgical robotics book [7] and from some papers [15, 21, 22]. These documents were produced by different authors, and vary greatly in the writing style: the procedure descriptions are essential and schematic in some cases, while longer sentences enriched with background information are used in others. The dataset consists of 20 descriptions of real-world procedures (taken as-is from the sources), from different surgical fields (urological, gynecological, gastrointestinal and thoracic). Regarding the book [7], we have arbitrarily selected without lack of generality a few (among many) of the sections describing surgical procedures (full section details on the dataset web-page). More precisely, we have only annotated those chapters and sections that, given their name (e.g. “Operational steps”), are expected to describe the procedure of a surgical intervention, leaving out evidently unrelated ones (e.g., “History of Robots and Robotic Surgery”). This because our goal is to identify the sentences in a procedure description that detail some of the surgical actions performed, automatically cleaning out all those sentences that are not-relevant for building a procedural workflow. Irrelevant sentences account for a substantial amount, as we will show later in the dataset statistics.

Each sentence in the selected procedure texts was manually annotated as *procedural* or *non-procedural*. To guide the annotation work and reduce labelling ambiguities (e.g., the same sentence may contain both procedural and non-procedural information), we defined some guidelines to be followed:

- *procedural*: a sentence describing at least one action by the robot or the human surgeon, being it an intervention on the body or the positioning of the robot;
- *non-procedural*: a sentence that does not include any indication of a specific surgeon action, but rather describes anatomical aspects, exceptional events that can occur during surgery and general indications that are not specific of a single step of the intervention.

The actual annotation of the 1958 sentences was performed by a single human annotator (M.Sc. with “C1” English language proficiency) with a 2-year experience in the robotic-surgical domain. The annotation of the whole dataset required approximately 65 working hours to the annotator. As frequently occurring with text classification tasks, the resulting annotated dataset is slightly unbalanced: $\sim$64% of all the sentences are classified as procedural, while the remaining $\sim$36% as non-procedural. That is, approximately one-third of the sentences in the collected text describing surgical intervention procedures does not describe concrete surgeon actions, and therefore these sentences are potentially not-relevant for deriving the intervention workflow.

As manual annotation is a rather subjective process, performed in our case by a single annotator, in order to assess the general adherence of the annotations produced with respect to the presented guidelines, we performed an inter-annotation agreement analysis: 98 sentences, approximately 5% of the overall dataset, were randomly sampled, respecting the procedural/non-procedural balancing of the dataset, and a second expert (Ph.D. with “C1” English language proficiency, computer science background) was asked to annotate them following the same guidelines. We obtained a Kappa coefficient of 0.93 which indicates an almost perfect level of agreement between the two annotators [12].

Our dataset is absolutely unique in literature and is freely available for research purposes. For a more detailed description of the dataset and requesting its download, we refer the reader to the dataset web-page.

### Preprocessing the dataset

In this paper, we tested different combinations of text normalization techniques in order to reduce the number of word forms in the original dataset and thus limiting noisy features. In particular, we lowercased each word, we replaced each number with a fixed placeholder, we removed punctuation, leading/ending white spaces, and stopwords. We also experimented combining these techniques with either lemmatization or stemming, but they turned out to be ineffective in our evaluation scenario.

### Classifiers

We frame the problem of automatically detecting procedural sentences in a written surgical intervention text as a sentence classification task. Many classification algorithms were proposed over the years, and to better assess the feasibility of our approach we experimented and compared the performance of different text classifiers, ranging from classical ML approaches to recent NN methods and Transformer-based approaches.

Given the reduced size of the dataset, for each model we applied the nested k*I-fold cross-validation protocol with k=10 and I=k-1=9. That is, the dataset is split into 10 sets. One by one, a set is selected as *test* set to assess the model performance, while the other 9 are used to fit the model (8 sets - a.k.a. *train* set) and determine the best hyperparameters^2^ (1 set - a.k.a. *validation* set), until all possible combinations have been evaluated. The model performance is then the average of the performances on each of the 10 test sets of corresponding model trained and tuned (according to the best hyperparameters) on the remaining 9 sets. This technique ensures that no data leakage can occur [5].

#### Classical ML approaches

We first analyzed some widely used classical ML methods, successfully applied for text classification: namely, *Random Forest* (c.f. [23]); *Linear Support Vector Machine* (c.f. [20]); *Multinomial Naïve Bayes* (c.f. [1]) and *Logistic Regression* (c.f. [18]).

These classifiers expect numerical feature vectors with a fixed size, rather than raw text of variable length [16], and therefore sentences have to be appropriately pre-processed. Specifically, for each term of a sentence in our dataset, we calculate a measure called *Term-Frequency, Inverse-Document-Frequency* (TF-IDF). A sentence is then represented as a vector, where the components correspond to the most frequent terms of the dataset, and the value in the components is the TF-IDF measure for that term of the sentence. The classifiers are then trained with these vectors.

#### FastText classifier

We then decided to test the effectiveness of the FastText Classifier [10] for the detection of procedural knowledge in written surgical text. FastText provides a library for efficient learning of word representations and sentence classification. It is widely used for numerous tasks, such as mail classification [25] and explicit content detection [19].

In FastText each word of a text is represented as a bag of character n-grams (a.k.a. “subwords”). Subwords allow taking into account the internal structure of the words when building the representation, capturing also morphological aspects of the words. They allow to better deal with languages with large vocabularies and rare words, allowing to compute word representations also for words never seen during the training phase. To perform the classification task, FastText adopts a multinomial logistic regression method, where each input sentence is encoded as a sentence vector, obtained by averaging the FastText word representations of all the words in the sentence.

#### Neural-network classifiers

We tested also some Neural-Network classifiers, that proved to be very effective in many different classification tasks and domains (e.g., [11, 14]):

- a 1D-CNN, i.e., a 1-Dimension Convolutional Neural-Network, one of the most widely used NN models for text classification;
- a Bi-LSTM (Bi-directional Long short-term memory) Neural-Network, an architecture exploiting memory support that is capable of capturing word dependencies inside sentences, even among far away words.

Given the possibility to build the FastText word embeddings separately from the FastText classifier, both neural approaches here considered were fed with the same sentence vectors used to train and evaluate the FastText classifier. This also allows us to draw a direct comparison between the efficient linear classifier implemented in FastText and the more advanced neural approaches.

#### Transformer-based classifiers

One of the most recent achievement in NLP was the release of Google’s BERT [6], an auto-encoding language model based on the multi-layer bidirectional Transformer encoder implemented in [26], that enables to achieve state-of-the-art performance on many NLP tasks^3^ such as question answering and language understanding. Differently from other word representation approaches (e.g., FastText), word vectors in BERT are *contextualized*, meaning that the embedding of a word will be different according to the sentence in which it is used. It is a pre-training approach (on the Masked Language Model task), that can be fine-tuned (even with a relatively small amount of data) on a specific task of interest, without having to rebuild the whole model from scratch. For our purposes, it means that we can fine-tune BERT for procedural sentences classification.

A possible limitation in the application of BERT for procedural sentence classification in surgical texts is that the language model is pre-trained on general domain texts (800M words from BooksCorpus and 2500M words from English Wikipedia), which are substantially different than the robotic-surgery documents we are working with, and this may negatively affect classification performance. To possibly mitigate this, we decided to evaluate also the impact of ClinicalBERT [3], a language model pre-trained on clinical notes and Electronic Health Records (EHR). While still different than surgical procedure descriptions, these texts are certainly closer to the robotic-assisted surgery domain than those used for training BERT.

In order to fine-tune BERT and ClinicalBERT for procedural sentence classification in robotic-assisted surgical texts, these pre-trained models have to be modified to produce a classification output (procedural/non-procedural). This is achieved by adding a classification layer on top of the pre-trained models, and then by training the entire model on our annotated dataset until the resulting end-to-end model is well-suited for our task. In details, for the sentence classification part, we actually use a single linear layer, similarly to what done in [3]. Note that some pre-processing of the dataset has to be performed in order to use its texts to fine-tune BERT and ClinicalBERT, such as word tokenization and index mapping to the tokenizer vocabulary, and fixed-length normalization (by truncation or padding) of all texts.

### Research questions and evaluation metrics

In this paper we investigate the following research questions:

1. Are the TF-IDF features fed to classic classification algorithms sufficient to detect procedural knowledge in surgical written texts? Is it necessary to resort to more sophisticated techniques of word embeddings and NNs? Do more modern and complex methods based on fine-tuning pre-trained language models outperform the other considered approaches?
2. Do some dataset balancing techniques positively impact the performances of procedural sentence classification?

To address these research questions, we compare the prediction of the various classifiers against some gold annotations (i.e. a set of sentences annotated with a procedural/non-procedural label). We do the comparison computing standard metrics adopted in binary classification tasks (c.f. [29]): Macro-Averaged Metrics, i.e., Precision (P), Recall (R), F-Score (F1); Weight-Averaged Metrics, i.e., w-Precision (wP), w-Recall (wR), w-F-Score (wF1); and, Accuracy (A).

## Results and discussion

**Table 1:** Classification performance of the tested methods

Method	Procedural			Non-Procedural			A	Macro			Weighted		
	P	R	F1	P	R	F1		P	R	F1	wP	wR	wF1
RandomForest	0.738	0.913	0.816	0.747	0.443	0.556	0.740	0.743	0.678	**0.686**	0.741	0.740	**0.721**
MultinomialNaïveBayes	0.717	0.965	0.823	0.852	0.344	0.491	0.737	0.785	0.655	0.657	0.767	0.737	0.701
LinearSVM	0.706	0.964	0.815	0.835	0.308	0.450	0.723	0.770	0.636	0.633	0.753	0.723	0.681
LogisticRegression	0.678	0.981	0.802	0.861	0.199	0.323	0.694	0.770	0.590	0.562	0.745	0.694	0.626
FastText	0.821	0.846	0.833	0.720	0.683	0.701	0.786	0.771	0.765	0.767	0.784	0.786	0.785
FastText[bal]	0.824	0.846	0.835	0.722	0.689	0.705	0.788	0.773	0.767	**0.770**	0.786	0.788	**0.787**
1D-CNN	0.889	0.834	0.861	0.742	0.821	0.780	0.829	0.816	0.828	0.820	0.835	0.829	0.831
1D-CNN[bal]	0.881	0.851	0.866	0.758	0.803	0.780	0.833	0.819	0.827	**0.823**	0.836	0.833	**0.834**
BiLSTM	0.894	0.896	0.895	0.820	0.817	0.818	0.867	0.857	0.856	0.857	0.867	0.867	0.867
BiLSTM[bal]	0.887	0.910	0.898	0.837	0.801	0.819	0.870	0.862	0.855	**0.859**	0.869	0.870	**0.869**
BERT	0.875	0.916	0.895	0.843	0.775	0.808	0.864	0.859	0.845	0.851	0.863	0.864	0.863
BERT[bal]	0.867	0.922	0.894	0.850	0.757	0.801	0.862	0.859	0.840	0.847	0.861	0.862	0.860
ClinicalBERT	0.886	0.915	0.900	0.845	0.797	0.821	0.872	0.866	0.856	**0.860**	0.871	0.871	**0.871**
ClinicalBERT[bal]	0.874	0.922	0.897	0.851	0.8771	0.809	0.866	0.862	0.846	0.853	0.865	0.866	0.865

“[bal]” indicates training on a 50–50 balanced dataset (upsampling)

Bold values indicate the highest values of the Macro-F1 and Weighted-F1 for each category of classification method considered

In the first four rows of   Table 1, we report the classification performance of the classical ML algorithms that exploit TF-IDF as features. The considered ML approaches based on TF-IDF have mediocre performance when used to solve this task. This fact could be due to the unbalanced dataset, which is difficult to be handled by standard ML algorithms. Classical ML approaches are often biased towards the majority class (F1 scores on the procedural class are substantially higher than on the non-procedural one), not considering the data distribution. In the worst case, minority classes are treated as outliers and ignored. Moreover, TF-IDF cannot account for the similarity between the words in a document since each word is independently presented as an index. Among the considered ML algorithms, Random-Forest obtains the highest F1 scores.

The fifth row of Table 1 summarizes the performance of the FastText classifier. All scores demonstrate that FastText obtains much higher classification performance than the best considered ML method (Ra-Fo). In particular, it provides an improvement of $10.56\%$ over Macro-F1 and $8.15\%$ over Weighted-F1.

We then fed the FastText word embeddings learned on the dataset to a 1D-CNN and a Bi-LSTM. The adoption of more complex classification models allows, in our task, to substantially improve performance, as confirmed by the seventh and ninth rows of the Table. The 1D-CNN gives an improvement on the Macro-F1 of $6.46\%$ and on the Weighted-F1 of $5.54\%$, and the Bi-LSTM contributes to an improvement on the Macro-F1 of $10.50\%$ and on the Weighted-F1 of $9.45\%$ with respect to FastText performance. The downside is the computational time: with the configurations used in the experiments, FastText is 8 times faster than the 1D-CNN and 40 times faster than the Bi-LSTM.

Finally, the eleventh and thirteenth rows of the Table show that it is possible to achieve high classification performance also using transformer-based pre-trained language models. In particular, ClinicalBERT performs slightly better than Bi-LSTM (+ 0.12% of Macro-F1 and + 0.22% of Weighted-F1), while BERT performs slightly worse than Bi-LSTM. As somehow expected, given the characteristics of the source material used for pre-training the model (c.f., Sect. 3.3.4), ClinicalBERT (pre-trained on clinical notes) performs better than BERT (pre-trained on Wikipedia and BooksCorpus). Computational wise, fine-tuning transformers-based models on our dataset is 4 times slower than training Bi-LSTM.

We also investigated whether it is possible to boost classification performance by balancing the dataset. More precisely, we have applied standard random over-sampling techniques (i.e., the addition of random set of copies of the minority class samples to the data) [4] to obtain a perfectly balanced (50% procedural / 50% non-procedural) training material, reassessing classification performance. Given the inadequate performance of classical ML algorithms, we have limited this analysis only to the three approaches that use subword word-embeddings as features and to transformers-based methods. As shown in the rows of Table 1 tagged with [bal], adopting upsampling techniques does not substantially improve classification results. Indeed, in the case of transformer-based models, balancing the dataset actually has some (limited) detrimental effects.

**Fig. 1:**
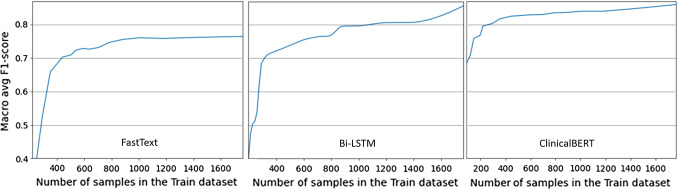
The trend of Macro-F1 of the FastText, Bi-LSTM and ClinicalBERT classifiers, obtained by varying the number of training samples

### Answer to research questions

Based on the reported results, we can answer RQ1 by stating that the considered ML methods fed with TF-IDF features do not provide a satisfactory solution to the problem. Using subword-enriched word embeddings fed to NNs allows to substantially improve the results, achieving overall good performance for the considered classification task (Bi-LSTM wF1 = 0.869 with balancing). Concerning pre-trained language models, a further (marginal) improvement is observed exploiting ClinicalBERT, while fine-tuning the general-domain BERT leads to lower classification performance than Bi-LSTM, showing that, for the considered task, more advanced (yet computationally demanding) techniques do not necessarily produce better results. Overall, the obtained results are certainly satisfactory, confirming the feasibility of automatically detecting procedural sentences in surgical intervention descriptions, and we believe there is still room for improvement, for example by enlarging the dataset.

Moreover, we cannot positively answer RQ2, as we experimentally observed that the adoption of upsampling techniques on the minority class does not substantially improve the performance for the detection of procedural knowledge.

### Considerations on the size of the dataset

We wondered if a dataset of 1958 sentences is large enough for the optimal training of learning approaches for this task, and if there is room to further improve the results by increasing the number of sentences in the dataset. To answer this question, we decided to study the evolution of the Macro-F1 when varying the size of the training dataset. Figure 1 (left) shows this analysis considering the FastText classifier. The curve tends to flatten out when reaching approximately 800 sentences in the Train dataset, thus possibly suggesting that the addition of more samples will unlikely yield substantially better performances. Figure 1 (center) shows the same analysis considering the Bi-LSTM classifier. The slope of the curve, especially approaching the whole size of the training dataset, is constantly increasing and does not flatten out. A similar trend, despite with a less prominent increase rate, is obtained for the same analysis on the classifier based on ClinicalBERT, shown in Fig. 1 (right). These trends somehow suggest that by increasing the number of samples of the dataset, classification performances might be further improved for these two methods.

Interestingly, the Figure also shows that ClinicalBERT’s fine-tuning on our dataset works very well, even for very limited-size datasets (F1>0.8 with just 400 samples).

## Conclusions and future works

The aim of this work was to introduce and investigate the problem, never tackled before, of the detection of procedural knowledge in written surgical intervention descriptions. The main contributions of this paper are: the proposal of framing the problem as a ML classification task; a novel, publicly accessible, annotated dataset that will allow other interested researchers to tackle this task and benchmark performances; the assessment of several classification methods that could be used for the detection of procedural knowledge in texts.

In particular, we tested the effectiveness of various ML algorithms operating on TF-IDF features, observing their poor performance. Better scores are achieved using the linear classification algorithm implemented by FastText, that works on subword enriched words-embeddings and finally, using the embeddings returned by FastText as the input features of some NN models (1D-CNN, Bi-LSTM). Finally, using ClinicalBERT to detect procedural sentences in robotic-surgical texts proved to be a good choice. From the experiments, it also emerged that balancing the number of class samples in the training dataset does not lead to a substantial performance boost.

We do not claim to have identified the best possible algorithm to tackle this problem nor to have identified the highest classification scores achievable. Our goal was indeed to provide a first assessment of the feasibility of the task using competitive methods. Indeed, we conjecture that the obtained results can still be improved, and we identified several directions for future work.

Concerning the dataset, enlarging it may be beneficial, also in light of the consideration reported toward the end of Sect. 4: to potentially speed up the annotation process, active learning could be worth investigating (i.e., collecting gold annotations by asking human evaluators to accept or correct the sentence classification predicted by the trained model).

In this work we tackled the procedural sentence detection task using information solely coming from the sentence to be classified. The integration of additional context-related (e.g., when a sentence is preceded by another “signaling” sentence or it appears in a bullet/numbered list) is worth investigating, in line with the recent work presented in [2].

The higher score of ClinicalBERT over BERT, given the nature of the training material used for building them, suggests that further improvement of the classification performance may be achieved by creating a BERT model specifically trained from scratch on surgical procedural language. Despite the quite demanding process, both in terms of computational power and time, this will also require the collection of a large amount of surgical texts to be used for training.

Finally, we remark that this work is a preparatory activity toward the long-term goal of extracting structured surgical intervention workflows from written procedural documents, a challenging and, to the best of our knowledge, never studied before task in the surgical domain.

## Data Availability

The source code will be released jointly with the dataset to interested researchers.

## Footnotes

1 Dataset web-page: https://gitlab.com/altairLab/spks-dataset.

2 For tuning hyperparameters, we either relied on built-in auto-tune functionalities (c.f., FastText) or the HyperBand algorithm [13].

3 Although some recent work claims that it does not really understand language [9].

## References

[1] Abbas M, Ali K, Memon S, Jamali A, Memon S, Ahmed A (2019). Multinomial Naive Bayes classification model for sentiment analysis. IJCSNS Int J Comput Sci Netw Secur.

[2] Agarwal S, Atreja, S, Agarwal V (2020) Extracting procedural knowledge from technical documents. arXiv preprint arXiv:2010.10156

[3] Alsentzer E, Murphy J, Boag W, Weng WH, Jin D, Naumann T, McDermott M (2019) Publicly available clinical BERT embeddings. In: Proceedings of the 2nd clinical natural language processing workshop, Association for Computational Linguistics, Minneapolis, Minnesota, USA, pp 72–78

[4] Batista G, Prati R, Monard MC (2004). A study of the behavior of several methods for balancing machine learning training data. SIGKDD Explor.

[5] Cawley GC, Talbot NLC (2010). On over-fitting in model selection and subsequent selection bias in performance evaluation. J Mach Learn Res.

[6] Devlin J, Chang MW, Lee K, Toutanova K (2018) Bert: pre-training of deep bidirectional transformers for language understanding. In: Proceedings of the 2019 conference of the North American chapter of the Association for Computational Linguistics: Human Language Technologies (NAACL-HLT)

[7] Fong Y, Woo Y, Hyung W, Lau C, Strong V (2018). The SAGES atlas of robotic surgery.

[8] Gupta A, Khosla A, Singh G, Dasgupta G (2018) Mining procedures from technical support documents. arXiv:1805.09780

[9] Jin D, Jin Z, Zhou JT, Szolovits P (2020). Is Bert really robust? A strong baseline for natural language attack on text classification and entailment. Proc AAAI Conf Artif Intell.

[10] Joulin A, Grave E, Bojanowski P, Mikolov T (2016) Bag of tricks for efficient text classification. arXiv preprint arXiv:1607.01759

[11] Kim Y (2014) Convolutional neural networks for sentence classification. In: Proceedings of the 2014 conference on empirical methods in natural language processing (EMNLP), pp. 1746–1751. Association for Computational Linguistics, Doha, Qatar

[12] Landis JR, Koch GG (1977). The measurement of observer agreement for categorical data. Biometrics.

[13] Li L, Jamieson K, DeSalvo G, Rostamizadeh A, Talwalkar A (2018). Hyperband: a novel bandit-based approach to hyperparameter optimization. J Mach Learn Res.

[14] Liu G, Guo J (2019). Bidirectional LSTM with attention mechanism and convolutional layer for text classification. Neurocomputing.

[15] Pardolesi A, Bertolaccini L, Brandolini J, Gallina F, Novellis P, Veronesi G, Solli P (2018). Four arms robotic-assisted pulmonary resection-right lower/middle lobectomy: how to do it. J Thorac Dis.

[16] Pedregosa F, Varoquaux G, Gramfort A, Michel V, Thirion B, Grisel O, Blondel M, Prettenhofer P, Weiss R, Dubourg V, Vanderplas J, Passos A, Cournapeau D, Brucher M, Perrot M, Duchesnay E (2011). Scikit-learn: machine learning in Python. J Mach Learn Res.

[17] Qian C, Wen L, Kumar A, Lin L, Lin L, Zong Z, Li S, Wang J, Dustdar S, Yu E, Salinesi C, Rieu D, Pant V (2020). An approach for process model extraction by multi-grained text classification. Advanced information systems engineering.

[18] Ramadhan WP, Astri Novianty STMT, Casi Setianingsih STMT (2017) Sentiment analysis using multinomial logistic regression. In: 2017 International conference on control. electronics, renewable energy and communications (ICCREC), pp 46–49

[19] Rospocher M (2020). Explicit song lyrics detection with subword-enriched word embeddings. Expert Syst Appl.

[20] Saigal P, Khanna V (2020). Multi-category news classification using support vector machine based classifiers. SN Appl Sci.

[21] Sarkaria IS, Rizk NP (2014). Robotic-assisted minimally invasive esophagectomy: the IVOR Lewis approach. Thorac Surg Clin.

[22] Savitt MA, Gao G, Furnary AP, Swanson J, Gately HL, Handy JR (2005). Application of robotic-assisted techniques to the surgical evaluation and treatment of the anterior mediastinum. Ann Thorac Surg.

[23] Shah K, Patel H, Sanghvi D, Shah M (2020). A comparative analysis of logistic regression, random forest and KNN models for the text classification. Augment Hum Res.

[24] Song S, Oh H, Myaeng SH, Choi S, Chun H, Choi Y, Jeong C, Zhong N, Callaghan V, Ghorbani AA, Hu B (2011). Procedural knowledge extraction on medline abstracts. Active media technology.

[25] Tahsin R, Mozumder MH, Shahriyar SA, Salim Mollah MA (2020) A novel approach for e-mail classification using fasttext. In: 2020 IEEE region 10 symposium (TENSYMP), pp 1392–1395

[26] Vaswani A, Shazeer N, Parmar N, Uszkoreit J, Jones L, Gomez A.N, Kaiser L.u, Polosukhin (2017) I.: attention is all you need. In: Guyon I, Luxburg UV, Bengio S, Wallach H, Fergus R, Vishwanathan S, Garnett R (eds) Advances in neural information processing systems, vol 30, Curran Associates, Inc

[27] Wambsganss T, Fromm H (2019) Mining user-generated repair instructions from automotive web communities. In: HICSS

[28] Yang H, Aguirre CA, De La Torre MF, Christensen D, Bobadilla L, Davich E, Roth J, Luo L, Theis Y, Lam A, Han TY, Buttler D, Hsu WH (2019) Pipelines for procedural information extraction from scientific literature: towards recipes using machine learning and data science. In: 2019 International conference on document analysis and recognition workshops (ICDARW), vol 2, pp 41–46

[29] Yang Y, Joachims T (2008). Text categorization. Scholarpedia.

[30] Zhang Z, Webster P, Uren V, Varga A, Ciravegna F (2012) Automatically extracting procedural knowledge from instructional texts using natural language processing. In: Proceedings of the eighth international conference on language resources and evaluation (LREC’12), European Language Resources Association (ELRA), Istanbul, Turkey, pp 520–527

